# Unravelling the Fate of Coronary Artery Disease in Patients Undergoing Valve Replacement for Severe Aortic Valve Stenosis

**DOI:** 10.31083/j.rcm2403068

**Published:** 2023-02-23

**Authors:** Lennert Minten, Johan Bennett, Keir McCutcheon, Christophe Dubois

**Affiliations:** ^1^Department of Cardiovascular Sciences, Katholieke Universiteit Leuven, 3000 Leuven, Belgium; ^2^Department of Cardiovascular Medicine, University Hospitals Leuven (UZLeuven), 3000 Leuven, Belgium

**Keywords:** coronary artery disease, aortic valve stenosis, percutaneous coronary intervention, transcatheter aortic valve implantation

## Abstract

Severe aortic valve stenosis is the most frequent valve pathology in the western world and approximately 50% of these patients have concomitant coronary artery disease (CAD). Revascularization of proximal obstructive CAD in patients undergoing surgical aortic valve replacement (SAVR) is common practice considered appropriate. However, the management of patients with CAD undergoing transcatheter aortic valve implantation (TAVI) is more controversial. Nevertheless, performing percutaneous coronary intervention (PCI) of significant (>70%) proximal coronary lesions is a widely adopted strategy, but robust supporting scientific evidence is missing. Some studies suggest that complex CAD with incomplete revascularization negatively impacts outcomes post-TAVI. As increasingly younger patients are undergoing TAVI, optimizing the long-term outcomes will become more important. Although PCI in TAVI patients is safe, no benefit on outcomes has been demonstrated, possibly due to an inadequate selection of prognostically important lesions for revascularization. A possible solution might be the use of coronary physiological indices, but these have their own limitations and more data is needed to support widespread adoption. In this review we provide an overview of current evidence on the outcomes after aortic valve replacement (AVR) and the evidence regarding revascularization in this population.

## 1. Introduction 

Coronary artery disease (CAD) and aortic valve stenosis (AS) have common risk factors and a shared pathogenesis, and therefore frequently co-exist in clinical practice [1]. In patients receiving surgical aortic valve replacement (SAVR), 40–65% of patients undergo concomitant coronary artery bypass grafting (CABG) and this percentage increases with age [2, 3]. In patients considered for transcatheter aortic valve implantation (TAVI), approximately 50% have obstructive CAD and half of these have multivessel disease with a reported mean Syntax score (SS) of 14 [4, 5, 6]. Both CAD and AS can present with dyspnea and angina, and their relative contribution to the complaints of the patient is often unclear [7]. Finally, AS can cause myocardial ischemia on its own, further complicating the assessment of CAD and the need for revascularization [8]. Significant CAD has been defined in the American guidelines as a minimal 70% reduction in diameter in a major coronary artery (50% in the left main coronary artery) and/or physiologically significance [9]. This guideline considers it is reasonable to revascularize these significant lesions both in patients undergoing SAVR (with concomitant CABG) or TAVI (with percutaneous coronary intervention (PCI) before TAVI) irrespective of anginal complaints, although only with a level C evidence. According to the latest European guidelines, in patients undergoing SAVR, CABG of lesions >70% stenosis is recommended (50% for left main) and CABG could be considered in lesions >50% stenosis [10]. Both recommendations are equally supported by a level C evidence. In patients undergoing TAVI, PCI should be considered in coronary artery diameter stenosis >70% in proximal segments, with again a level C evidence [10]. These weak recommendations underscore the level of uncertainty regarding the best way to assess and select CAD for potential treatment in this context. A strategy using coronary physiology indices has been proposed to improve lesion selection for revascularization and clinical outcomes, but such approach needs further validation in patients with severe AS [11, 12]. In this focused review, we describe the impact of CAD on clinical outcomes in patients undergoing aortic valve replacement (AVR) for AS, we depict the available methods to assess lesion severity and provide an overview of current available evidence on revascularization.

## 2. Impact of CAD on Outcomes 

### 2.1 Patients Undergoing SAVR

Current evidence suggests that patients with CAD and AS have worse clinical outcomes after AVR. A large systematic review showed that patients with CAD had a higher risk of early mortality after valve replacement [13]. Another retrospective analysis showed that omitting revascularization in these patients is an independent predictor for early mortality [14]. Moreover, Cox regression analysis identified CAD as a determinant of late mortality after hospital discharge.

### 2.2 Patients Undergoing TAVI

In contrast with SAVR, observational studies assessing the association of CAD and outcomes post-TAVI have provided heterogenic results. These studies show CAD to have either no, a partial (only severe/complex lesions) or a systematic negative prognostic effect on short term outcomes after TAVI. Even two large meta-analyses on this topic revealed conflicting results [6, 15]. One showed that the presence of CAD did not affect 30-day outcomes after TAVI, but demonstrated a significant negative effect of CAD on survival one year after the procedure [15]. The other suggested that the presence of CAD did not impact on 30-day or one year mortality after TAVI [6]. However, the presence of severe CAD (defined by a SS >22) in this study did result in a higher one-year mortality after TAVI. Conflicting results in observational data may be explained by differences in the definition used (history of CAD vs coronary lesions at the time of TAVI), the absence of systematic assessment of lesion severity (angiography-guided or using coronary pressure indices) or the lack of objective scoring of anatomical complexity (e.g., SS). Moreover, when a SS was calculated, different cut-off points to define severe CAD appear to have been used. Hence, whether the presence of CAD is simply a marker of atherosclerosis burden and other co-morbidities or rather represents an independent risk factor for worse outcomes after TAVI, remains unclear at this point. Importantly, current meta-analyses merely reported outcomes until 1 year after TAVI. It is not unreasonable to expect that the presence of significant CAD might need more time to exert its impact on clinical outcomes, underscoring the need for long-term follow-up studies. Further emphasizing this point, a recent prospective study showed that CAD-complexity was an important determinant of long-term outcomes up to five years post-TAVI, with the most complex CAD-group having the worst prognosis [16].

## 3. Assessment of CAD in Patients with AS

### 3.1 Non-Invasive Assessment 

There are multiple non-invasive methods for the assessment of the functional severity of CAD, by investigating the perfusion of the heart during stress. Stress testing is not routinely advised in current guidelines for valvular heart disease [9, 10], mainly due to difficulties in interpreting the cause of hypoperfusion if it is seen, due to severe changes in coronary hemodynamics in AS, and due to some concerns about safety in this population. Nevertheless, a number of smaller studies have shown promising results. Perfusion cardiac magnetic resonance imaging (MRI) and positron emission tomographic (PET) have shown to be safe in AS patients, however their ability to identify flow limiting coronary lesions needs still to be validated [17, 18, 19]. Stress transthoracic echocardiography (TTE) in AS patients showed good specificity and moderate sensitivity to detect >50% narrowing on invasive angiography [20]. Single-photon emission computed tomography (SPECT) myocardial perfusion imaging has found to be safe and predict significant CAD with a good sensitivity and specificity in patients with AS [21, 22, 23, 24]. It also showed a good correlation with invasive coronary hemodynamic indices of CAD (Fractional flow reserve (FFR) and instantaneous wave-free ratio (iFR)) [25, 26].

From an anatomical perspective, cardiac computed angiography (CTA) has successfully been used as an alternative to invasive coronary angiography (CA) during the pre-TAVI work-up [27]. Supporting this approach are numerous studies showing a very high sensitivity (89–99%) and negative predictive value (90–96%) compared with CA in this setting for the detection of significant coronary stenosis [28, 29, 30, 31, 32, 33, 34]. The disadvantage is a relative low specificity (37–91%) and positive predictive value (59–87%), although a broad range in values has been reported. Overall, CTA has a similar sensitivity but a lower specificity in severe AS patients when compared to patients without AS, probably in part due to the higher burden of coronary calcium in AS [35, 36]. Combining coronary CTA analysis with valve sizing and assessment of vascular access during work-up might decrease the number of CA performed by up to 37%, and this percentage may further decrease as younger patients with a lower risk profile are being selected for TAVI [36]. Moreover, CTA may offer evaluation of the functional severity of lesions on top of anatomical information, with promising modalities such as CT-derived FFR. A study in AS patients showed that this tool was safe and feasible in this population, however with a moderate sensitivity (74%) and specificity (78%) and with a diagnostic accuracy of 77% compared to invasive FFR [37]. It is the view of the authors that CTA can be used during the pre-SAVR/TAVI workup in severe AS patients without angina, mainly to avoid unnecessary CA in relatively young low-risk patients with a low pre-probability of CAD. In patients with a very high coronary risk profile, history of CAD or active angina the performance of CA would remain standard practice.

### 3.2 Angiographic Anatomical Assessment 

Studies assessing CAD in patients with severe AS have used a luminal narrowing of ≥50% [38, 39, 40, 41] or ≥70% [42, 43] during invasive CA to define significant disease. Visual assessment of lesion severity carries high inter- and intra-observer variability, and quantitative coronary angiography (QCA) may therefore be the preferred method [44]. However, there are some notable disadvantages: Firstly, there is no data showing that PCI guided by QCA for stable lesions in patients without AS offers any clinical benefit. Secondly, older studies have shown that coronary diameters might increase as AS progresses with a reversed effect after aortic valve replacement [45]. This might pose a problem in interpretation, as QCA before and after TAVI may change. Lastly, severe coronary artery tortuosity and eccentric lesions with heavy calcification, both fairly common in the AS population, create problems for interpretation and decrease measurement accuracy (especially in 2D QCA) [46].

Building further on QCA imaging, quantitative flow ratio (QFR) is a technique integrating functional relevance of a coronary stenosis without the use of Adenosine or a pressure wire. During angiography the flow of contrast is analyzed and 3D QCA information is computed to estimate a pressure loss over a given lesion. In a recent study in patients with AS, QFR had a better diagnostic performance than angiography alone to assess FFR-based significance of a lesion [47]. However, diagnostic accuracy decreased considerably when the aortic valve area (AVA) was smaller than 0.80 ${\mathrm{cm}}^{2}$ and especially when it was smaller than 0.60 ${\mathrm{cm}}^{2}$. This highlights the importance of altered hemodynamics in severe AS patients [48].

Alternatively, intravascular imaging using ultrasonography (IVUS) or optical coherence tomography, while providing more detailed anatomical information, has a limited role in the decision to revascularize in daily practice, and no studies are available in the AS population. However, for the treatment of left main coronary artery (LMCA) stenoses, a cut-off of >6.0 ${\mathrm{mm}}^{2}$ for the minimal luminal area (MLA) measured with IVUS has been proposed for safely deferring revascularization of the LMCA and this cut-off was clinically validated in a non-AS population [49, 50]. There are no immediate reasons to suspect this cut-off would be different in AS patients, however, population specific validation would be ideal before widespread adoption in clinical practice. Lower MLA cut-off values have been proposed and validated with FFR, however, caution should be used since these studies were performed only in Asian populations and a lower sensitivity and negative predictive value suggest significant LMCA stenosis might be missed using a lower cut-off [51, 52].

Finally, from a more global perspective, anatomical scoring systems can be used to further describe the extent and complexity of CAD in the individual patient. Studies in AS patients have used the Duke Myocardial Jeopardy score [53] or the synergy between percutaneous coronary intervention with taxus and cardiac surgery – score (SYNTAX-score, SS) [54]. Some studies showed that patients with a high baseline SS or high residual SS had worse outcomes after TAVI in comparison with patients with less complex disease [16, 44, 55], indicating that these scores can potentially be used for risk stratification in patients with AS.

### 3.3 Pressure Wire Assessment 

Angiographic assessment of luminal narrowing is a poor predictor of coronary hemodynamic physiological indices, such as coronary flow reserve (CFR), FFR, iFR and resting flow ratio (RFR). The blood flow through a coronary artery and its fractional decrease over a lesion are also dependent on other physiological and anatomical factors besides luminal narrowing, such as the length of the lesion and microvascular function [56]. There is data supporting the use of these indices in stable CAD and the FAME-2 trial supports performing PCI in lesions with FFR <0.8 [12, 57, 58]. Therefore it is advised to support a decision to revascularize intermediate to severe stable lesions on a coronary hemodynamic assessment [59, 60]. However, importantly, severe AS patients have been systematically excluded from these trials due to concerns related to the effect of valve stenosis and the resulting LV hypertrophy on the possibility of causing falls negative or positive results. Nevertheless, the potential importance of these indices in this population has been illustrated in a retrospective study that suggested better outcomes for AS patients when comparing FFR-guided PCI with angiography-guided PCI [61]. Future studies will provide more data on the effect of valve replacement on coronary hemodynamics in patients with severe AS [62].

## 4. Revascularization 

### 4.1 Revascularization in Surgical AVR 

Although patients who undergo combined AVR with CABG have higher unadjusted mortality, this difference is no longer present after propensity matching [63, 64]. Two retrospective studies showed that patients with significant AS and CAD undergoing SAVR with CABG had a significantly reduced early and late mortality when compared with SAVR alone [14, 65]. Therefore concomitant revascularization of coronary lesions >70% is currently advised, mostly in the form of CABG. However, a hybrid approach with PCI, could be considered as an alternative [66, 67].

### 4.2 Revascularization in TAVI 

Treatment of stable CAD with PCI in non-AS patients is controversial, and should potentially be viewed as mainly a symptomatic treatment, as several prospective randomized studies have not showed a clear prognostic benefit [68, 69, 70]. This becomes even more complex in the AS population, especially those who are elderly and frail. Revascularization with PCI pre-TAVI of every proximal coronary lesion with ≥70% narrowing is currently not supported by strong scientific evidence as documented in the latest guidelines [9, 10]. However, as TAVI indications are expanding to younger and lower-risk patients, the correct assessment and treatment of CAD is key to optimize outcomes and quality of life of these patients. It has been shown that more complex CAD is associated with more myocardial injury (on the basis of serum troponins) post-TAVI [71, 72]. Performing PCI of these lesions has shown to be safe as short-to-intermediate-term outcomes among patients with CAD that either did or did not undergo PCI have been found to be comparable in numerous observational studies (Table 1, Ref. [16, 41, 43, 44, 73, 74, 75, 76, 77, 78]). Nevertheless, comparable clinical outcomes can also be seen as evidence that revascularization of lesions ≥70% pre-TAVI is not absolute necessary. Ten percent of patients included in randomized studies comparing SAVR (+/– CABG) versus TAVI (+/– PCI) in low risk patients received revascularization [79, 80, 81, 82]. These trials showed superiority or non-inferiority for the transcatheter arm for the combined end-point (stroke or death) at medium-term follow-up, indirectly supporting TAVI with PCI. Unfortunately, only one trial reported data on the subset of patients that underwent PCI or CABG (90 patients in total) [82]. A non-significant difference could be seen for the chance of rehospitalization/stroke/death between the groups favoring PCI (9.4% vs 12.1%, HR: 0.77; 95% CI: 0.20–2.98) [82]. Importantly, these randomized trials excluded patients with complex CAD (SS >22 or 32) and patients with significant left main disease.

**Table 1.** **Overview of studies investigating the impact of stable CAD and peri-TAVI revascularization on all-cause mortality after TAVI**.

Study	Design	Population	Follow-up time	Outcome	Result
Wenaweser *et al*. 2011 [73]	Single-centre prospective registry	197 TAVI	2 years	All-cause Mortality	No difference (*p* = 0.96)
		vs 59 TAVR + PCI			
Abdel-Wahab *et al*. 2012 [41]	Single-centre retrospective registry	70 TAVI	3 years	All-cause Mortality	No difference (*p* = 0.36)
		vs 55 TAVI + PCI			
Codner *et al*. 2013 [74]	Single-centre prospective registry	117 TAVI	2 years	All-cause Mortality	No difference (*p* = 0.67)
		vs 36 TAVI + PCI			
Abramowitz *et al*. 2014 [43]	Single-centre prospective registry	105 TAVI (without CAD)	3 years	All-cause Mortality	No difference (*p* = 0.68)
		vs 83 TAVI (with CAD)			
		vs 61 TAVI + PCI			
Khawaja *et al*. 2015 [44]	Single-centre retrospective registry	68 TAVI (with CAD)	1 year	All-cause Mortality	No difference (*p* = 0.918)
		vs 25 TAVI + PCI			
Snow *et al*. 2015 [75]	Multicentre prospective registry	2005 TAVI without historical PCI	5 years	All-cause Mortality	No difference (*p* = 0.81)
		vs 363 TAVI with historical PCI			
		vs 169 TAVI + hybrid PCI			
		vs 169 TAVI + PCI			
Huczek *et al*. 2016 [76]	Multicentre retrospective registry	434 isolated TAVI (without CAD)	30 days	All-cause Mortality	No difference (*p* = 0.098)
		vs 293 isolated TAVI (with CAD)			
Chakravarty *et al*. 2016 [77]	Multicentre retrospective registry	128 isolated TAVI	1 year	All-cause Mortality	No difference (HR: 1.09; 95% CI: 0.50–2.39; *p* = 0.83)
		vs 128 TAVR + LM PCI			
		(1:1 case-control matched)			
Millan-Iturbe *et al*. 2017 [78]	Single-centre prospective registry	720 isolated TAVI (without CAD)	9 years	All-cause Mortality	No difference (*p* = 0.229)
		vs 88 TAVI (with CAD)			
		vs 136 TAVI + PCI			
Minten *et al*. 2022 [16]	Single-centre prospective study	239 isolated TAVI	5 years	All-cause Mortality	No difference (*p* = 0.162)
		vs 107 TAVI + PCI			

CAD, coronary artery disease; 95% CI, 95% confidence interval; CR, complete revascularization; HR, hazard ratio; IR, incomplete revascularization; LM, left mainstem coronary artery; MACCE, major adverse cardiac and cerebrovascular events; PCI, Percutaneous coronary intervention; rSS, residual Syntax score; SS, Syntax-score; TAVI, Transcutaneous aortic valve implantation.

In patients with stable CAD and ischemia without significant valvular disease, the ISCHEMIA trial confers limited to no influence on early invasive revascularization strategy on outcomes [70]. Nevertheless, AS patients with a very high burden or very complex CAD (represented by a high SS-score) may have better outcomes when revascularized. Data to support this comes from observational studies that analyzed completeness of revascularization. Several studies have shown a correlation between incomplete revascularization (high residual SS) and worse clinical outcomes such as increased mortality or major adverse cardiac or cerebrovascular events (MACCE) supporting PCI in the peri-TAVI period [83, 84, 85, 86, 87, 88]. However, other studies could not find an association between incomplete revascularization and clinical events [38, 89, 90, 91, 92, 93]. Recently, two important papers regarding this topic were published. One prospective study with 5-year follow-up showed there was no benefit of (complete) revascularization for stable CAD in TAVI patients [16]. A large retrospective registry among TAVI patients with significant stable CAD showed no benefit of complete myocardial revascularization to reduce the risk of all cause death at 2 years [94]. Limitations in combining the results of these studies lie in differences in the definition of incomplete revascularization, follow-up times and comorbidities, and overall small patients numbers in the cohorts studied (Table 2, Ref. [16, 38, 83, 84, 85, 86, 87, 88, 89, 90, 91, 92, 93, 94]).

**Table 2.** **Overview of studies investigating the impact of completeness of revascularization in the peri-TAVI period**.

Study	Design	Population	Follow-up time	Outcome	Result
Ussia *et al*. 2013 [89]	Multicentre prospective registry	92 TAVI + no PCI	1 year	All-cause mortality	No difference (*p* = 0.807)
		88 TAVI + IR		MACCE	No difference (*p* = 0.594)
		95 TAVI + CR			
Van Mieghem *et al*. 2013 [38]	Single-centre prospective study	124 TAVI + IR	1 year	All-cause mortality	No difference (*p* = 0.85)
		139 TAVI + CR			
Stefanini *et al*. 2014 [83]	Single-centre prospective registry	TAVI + PCI both groups:	1 year	MACCE	High residual SS = higher risk (RR: 1.92; 95% CI: 1.02–3.61; *p* = 0.042)
		- 192 low residual SS (0–14)			
		- 95 high residual SS (>14)			
Kleczynski *et al*. 2016 [84]	Single-centre prospective registry	16 TAVI + IR	1 year	All-cause mortality	IR = higher mortality (HR: 10.86; 95% CI: 3.72–31.73; *p *< 0.001)
		85 TAVI + CR			
Paradis *et al*. 2017 [90]	Multicentre retrospective registry	TAVI all groups:	1 year	MACCE	No difference (*p* = 0.16)
		- 82 No CAD			
		- 17 low residual SS (0–7)			
		- 37 high rSS (≥8)			
Shamekhi *et al*. 2017 [85]	Single-centre prospective study	TAVI all groups:	3 years	All-cause mortality	Univariate analysis: higher residual SS = increased mortality (*p* = 0.01)
		- 229 no CAD			Multivariate analysis: no significant effect of rSS
		- 140 low residual SS (0–3)			
		- 205 high residual SS (>3)			
Witberg *et al*. 2017 [86]	Multicentre retrospective registry	TAVI all groups:	5 years	All-cause mortality	High rSS = higher mortality (HR: 1.72; 95% CI: 1.051–2.814; *p* = 0.031)
		- 817 no CAD			
		- 331 low residual SS (0–8)			
		- 122 high residual SS (>8)			
Li *et al*. 2019 [91]	Single-centre retrospective registry	TAVI + PCI in all groups:	3 years	All-cause mortality	No difference (*p* = 0.40)
		- 144 CR		MACCE	No difference (*p* = 0.18)
		- 151 major IR			
		- 29 minor IR			
López Otero *et al*. 2019 [92]	Single-centre retrospective registry	TAVI + PCI in all groups:	3 years	All-cause mortality	No difference (*p* = 0.605)
		- 56 CR (rSS = 0)		MACCE	No difference (*p* = 0.866)
		- 85 RCR (rSS = 1–7)			
		- 46 IR (rSS ≥8)			
Saia *et al*. 2019 [93]	Single-centre retrospective registry	TAVI + PCI in both groups:	5 years	Cardiovascular mortality	No difference (*p* = 0.25)
		- 138 CR			
		- 153 IR			
Landt *et al*. 2019 [87]	Single-centre retrospective registry	TAVI + PCI in both groups:	1 year	All-cause mortality	CR = lower mortality (HR: 0.450; 95% CI: 0.218–0.926, *p* = 0.030)
		- 129 CR (rSS = 0)			
		- 78 IR (rSS >0)			
Faroux *et al*. 2020 [88],	Multicentre retrospective registry	TAVI + PCI in both groups:	2 years	MACCE	CR = lower MACCE (HR: 0.77; 95% CI: 0.63–0.95, *p* = 0.014)
		- 889 CR			
		- 308 IR			
Minten *et al*. 2022 [16]	Single centre prospective study	TAVI + PCI in all groups:	5 years	All-cause mortality	No difference (*p* = 0.678)
		- 66 RCR (rSS = 1–7)		Cardiovascular mortality	No difference (*p* = 0.361)
		- 41 IR (rSS ≥8)			
Costa *et al*. 2022 [94]	Multi-centre retrospective registry	Stable CAD in TAVI:	2 years	Cardiovascular death	No difference (*p* = 0.63)
		- 657 CR		MACCE	No difference (*p* = 0.94)
		- 287 IR			
		- 370 no revascularisation			

CAD, coronary artery disease; 95% CI, 95% confidence interval; CR, complete revascularization; HR, hazard ratio; IR, incomplete revascularization; LM, left mainstem coronary artery; MACCE, major adverse cardiac and cerebrovascular events; PCI, Percutaneous coronary intervention; rSS, residual Syntax score; SS, Syntax-score; TAVI, Transcutaneous aortic valve implantation.

**Fig. 1.**
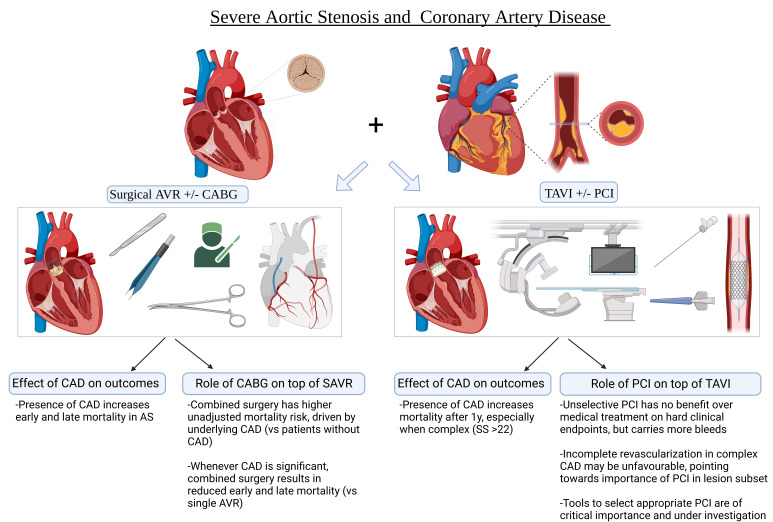
**Central Figure with overview**.

To date, only one randomized clinical trial comparing TAVI with medical therapy vs TAVI with PCI in patients with severe AS and CAD has been performed [95]. CAD was defined as stenosis severity of >70%, 235 patients were enrolled and PCI was angiography-guided. At one-year there was no difference in the combined endpoint of rehospitalization or death between the medical and PCI group (44.0% vs 41.5% resp., *p* = 0.067). Furthermore, there were no differences in rates of acute kidney injury, myocardial infarction or stroke. Importantly, patients undergoing PCI presented significantly more all-cause bleeds (28.4% vs 44.5%, *p* = 0.02) after one year. Of note, this trial did not reach its recruitment target (310 patients), non-inferiority of the primary end-point was not met and the study population had low rates of reported angina (70% of the patients reported Angina Canadian Cardiovascular Society class 0). A overview of the current evidence regarding the effect of CAD on outcomes after aortic valve replacement and the effect of revascularization can be found in Fig. 1.

Ideally, additional tools are needed to select patients benefiting most from PCI at the time of TAVI. Unfortunately, the invasive assessment of coronary hemodynamics by using indices such as FFR, iFR, and RFR cannot simply be extrapolated from patients with stand-alone stable CAD to a population with AS [48]. Severe AS induces dramatic changes in coronary physiology that are still incompletely understood. Moreover, it is unclear which index to use since it is expected that severe AS and valve replacement impact differently on these indices [96]. When looking at outcomes, one observational study compared patients who underwent angiography versus physiology-guided PCI before TAVR [61]. In this study, patients in whom the decision was based on physiological assessment had better survival free from MACCE at two years follow-up (HR: 0.40; 95% CI: 0.20–1.00, *p* = 0.035). More studies to investigate the role of coronary physiological in AS are currently underway [62].

### 4.3 Timing of Revascularization 

In case of acute cardiac symptoms (such as chest pain and dyspnea), raised troponin levels and ECG changes, the difficult differential between an acute coronary syndrome and acute decompensated AS should lead to the predominate cause of decompensation being treated first [97]. However, most decisions in this population are made in an elective setting.

For patients going to SAVR, CABG should be performed during the same procedure for obvious reasons. In contrast, patients receiving TAVI can undergo revascularization before, during or after valve implantation, and several considerations can be made in this respect.

Performing PCI before TAVI has the theoretical disadvantage of inflicting multiple hospital admissions and invasive cardiovascular procedures to the patient with repeated risk for contrast induced kidney injury, while increasing dual antiplatelet-related bleeding risk following TAVI. The benefits of this strategy include the potential reduction of ischemia during TAVI, in case of severe lesions with high-risk features or left main stem disease, especially when rapid pacing is required [98]. Moreover, the PCI-first strategy will maintain optimal coronary access, allowing optimal guide catheter support for more complex revascularization. It remains unclear, however, how long the time interval between PCI and TAVI should be. One study showed that there was no significant difference in mortality at 2 year follow-up between PCI within one month or more than one month prior to TAVI [99]. Nevertheless, the group with the PCI closer to the valve procedure had significantly more bleeding and minor vascular complications, suggesting a potential benefit of leaving enough time between both procedures.

Performing PCI and TAVI in one procedure is feasible and limits the number of hospital admissions and invasive cardiovascular procedures [100, 101]. However, the higher volume of contrast medium administered in these combined interventions carries an increased risk for acute kidney injury, especially when considering complex CAD interventions in frail patients. Furthermore, the results of a large registry suggest that patients undergoing PCI during the same admission as TAVI had a higher rate of complications and mortality [102].

There is general agreement that overall, in patients with severe AS and CAD, the severity of the valve disorder is driving the symptoms and risk, and PCI should only be considered for severe proximal lesions in vessels supplying a large myocardial territory [9]. Deferring PCI until after TAVI, to observe how symptoms (and coronary indices such as FFR and RFR) evolve, seems like a valid strategy, especially in equivocal lesions [103]. This is supported by previously mentioned studies showing no short-term outcome benefit of PCI. Moreover, although patients with complex (CAD) had worse outcomes in a recent study, this difference only started to appear after a few years [16]. TAVI operators should however take into account the impact of the valve procedure on future coronary access as in some cases access may become technically challenging depending on the anatomy of the aortic root and the valve type used [104]. While it has been reported that PCI after TAVI has a high success rate for all available transcatheter valves [105, 106], modifications in PCI and TAVI technique are sometimes necessary [107, 108]. In this respect, the use of the Evolut R/PRO valve, the interaction of the valve with the sinus of Valsalva and the mean valve implantation depth have been identified as independent predictors for difficult coronary access post-TAVI [109].

## 5. Future Perspectives 

The challenge remains to identify which patients and coronary lesions may benefit from myocardial revascularization at the time of AVR in the setting of severe AS. While non-invasive functional imaging, such as CT-based FFR looks promising, its potential role, even in a context without AS, needs to be finetuned. However, invasive assessment of coronary physiology by means of FFR and non-hyperemic pressure ratios (NHPRs) has become standard practice in cathlabs, and decision algorithms for revascularization should now be validated or adapted in the context of AS. Currently, several smaller or larger scale studies are recruiting patients in this field, focusing on either mechanistic understanding of physiologic variables impacted by AS, or rather pure clinical outcomes (Table 3, Ref. [62, 110, 111, 112, 113, 114, 115]). Ultimately, randomized controlled trials, will be needed to answer remaining questions. As a matter of fact, the COMPLETE TAVR trial (NCT04634240) is comparing medical therapy versus complete revascularization in patients undergoing TAVI. The TAVI-PCI (NCT04310046) study is trying to determine the ideal timing for physiology-guided revascularization relative to the TAVI. Further studies regarding gender differences in regard to CAD in patients undergoing TAVI will also be important [116].

**Table 3.** **Future and ongoing studies on CAD physiological assessment and treatment in TAVR patients**.

Study	Design	Population	Recruitment target	Description	Primary outcome	Completion date
**FORTUNA **[110] (NCT03665389)	Single centre, prospective open-label study	TAVI patients with moderate- severe CAD	25	CT-based FFR, FFR and iFR pre-TAVR and FFR and iFR post-TAVR	Comparison between CT based FFR and iFR/FFR	2023
**FAVOR IV-QVAS **[111] (NCT03977129)	Multicentre, randomized control trial	AS patients undergoing valve surgery + moderate to severe CAD	792	Angiography guides vs QFR guided revascularization	Composite endpoint: all-cause mortality, MI, stroke, unplanned revascularization, kidney injury requiring dialysis at 30 days	2026
**COMIC-AS **[62] (NCT04420325)	Multicentre, prospective cohort study	AS patients undergoing TAVI or SAVR + moderate to severe CAD	100	FFR, RFR, CFR and IMR with SPECT myocardial perfusion imaging pre-(T)AVR and FFR, RFR, CFR and IMR immediately and 6 months after TAVI	Change in FFR and RFR, correlation between indices and non-invasive imaging at 6 months	2024
**TCW **[112] (NCT03424941)	Multicentre, open-label, non-inferiority randomized controlled trial	AS patients undergoing SAVR/TAVI with multivessel CAD	328	CABG + SAVR vs FFR-guided PCI +TAVI	Composite endpoint: mortality, MI, disabling stroke, major bleeding, valve re-intervention or need for target lesion revascularization at 1 year	2024
**FAITAVI **[113] (NCT03360591)	Single centre, open-label, randomized controlled trial	AS patients undergoing TAVI with moderate to severe CAD	320	Angiography guided versus physiology guided PCI	Composite endpoint: all-cause mortality, MI, stroke, major bleeding and target lesions revascularization at 1 year	2024
**NOTION-3 **[114] (NCT03058627)	Multicentre, open-label, randomized controlled trial	AS patients undergoing TAVI, with one significant coronary lesion (FFR ≤0.80 or >90% diameter stenosis)	452	TAVI + FFR-guided complete revascularization vs TAVI + medical management of CAD	Composite endpoint: all cause death, myocardial infarction, or urgent revascularization at 1 year	2027
**TAVI-PCI **[115] (NCT04310046)	Open-label, randomized controlled trial	AS patients undergoing TAVI and PCI for CAD	986	iFR-guided revascularization: performed 1–45 days before versus 1–45 days after TAVI	Composite endpoint: All-cause mortality, MI; ischemia driven revascularization, rehospitalization, major bleeding at 1 year	2028

CABG, Coronary Artery Bypass Grafting; CAD, coronary artery disease; CFR, coronary flow reserve; CT, computed tomography; FFR, fractional flow reserve; iFR, instantaneous wave-free ratio; IMR, index of microvascular resistance; MI, myocardial infarction; QFR, Quantitative flow ratio; RFR, resting full-cycle ratio; SAVR, surgical aortic valve replacement; SPECT, Single photon-emission computed tomography; TAVI, transcutaneous aortic valve implantation.

## 6. Conclusions 

Patients with CAD and severe AS represent a frequently-encountered clinical entity in daily practice. It appears that (complex) CAD independently negatively influences the outcomes after AVR and so deserves particular attention. Although important, the ideal methods to assess and treat CAD in this population remain unclear. Some data suggest complete revascularization might benefit these patients but many studies fail to show a beneficial effect of angiography-guided PCI in this population. Severe AS induces severe coronary hemodynamic changes that make the physiological assessment of lesions severity challenging. Nevertheless, this field is advancing rapidly and several large clinical trials are actively recruiting and will significantly improve our understanding of CAD in the setting of severe AS.
